# Palmitoyl Protein Thioesterase 1 Is Essential for Myogenic Autophagy of C2C12 Skeletal Myoblast

**DOI:** 10.3389/fphys.2020.569221

**Published:** 2020-10-15

**Authors:** Hyeong Rok Yun, Yong Hwa Jo, Jieun Kim, Ngoc Ngo Yen Nguyen, Yoonhwa Shin, Sung Soo Kim, Tae Gyu Choi

**Affiliations:** ^1^Department of Biomedical Science, Graduate School, Kyung Hee University, Seoul, South Korea; ^2^Biomedical Science Institute, Kyung Hee University, Seoul, South Korea; ^3^Department of Biochemistry and Molecular Biology, School of Medicine, Kyung Hee University, Seoul, South Korea

**Keywords:** palmitoyl protein thioesterase 1, mitochondrial reactive oxygen species, autophagy, mammalian target of rapamycin complex, muscle differentiation

## Abstract

Skeletal muscle differentiation is an essential process for the maintenance of muscle development and homeostasis. Reactive oxygen species (ROS) are critical signaling molecules involved in muscle differentiation. Palmitoyl protein thioesterase 1 (PPT1), a lysosomal enzyme, is involved in removing thioester-linked fatty acid groups from modified cysteine residues in proteins. However, the role of PPT1 in muscle differentiation remains to be elucidated. Here, we found that PPT1 plays a critical role in the differentiation of C2C12 skeletal myoblasts. The expression of PPT1 gradually increased in response to mitochondrial ROS (mtROS) during muscle differentiation, which was attenuated by treatment with antioxidants. Moreover, we revealed that PPT1 transactivation occurs through nuclear factor erythroid 2-regulated factor 2 (Nrf2) binding the antioxidant response element (ARE) in its promoter region. Knockdown of PPT1 with specific small interference RNA (siRNA) disrupted lysosomal function by increasing its pH. Subsequently, it caused excessive accumulation of autophagy flux, thereby impairing muscle fiber formation. In conclusion, we suggest that PPT1 is factor a responsible for myogenic autophagy in differentiating C2C12 myoblasts.

## Introduction

Skeletal muscle differentiation is a highly coordinated multistep process, which generates myotubes. Muscle differentiation proceeds through cell cycle arrest of myoblasts and requires expression of multiple muscle-specific genes, leading to the differentiation of myoblasts to mature muscle fibers (Barbieri and Sestili, 2012). Myogenic differentiation requires the expression of a group of basic helix-loop-helix muscle regulatory transcription factors, such as muscle regulatory factors (MRFs), Myf5, MyoD, myogenin, and MRF4 (Molkentin and Olson, 1996; Arnold and Winter, 1998).

Palmitoyl protein thioesterase 1 (PPT1) is a lysosomal hydrolase that catalyzes the cleavage of thioester linkages in palmitoylated peptides or proteins, which facilitates the degradation of these polypeptides (Camp and Hofmann, 1993; Zeidman et al., 2009). Palmitoylation is one of the most important post-transcriptional protein modifications, which is involved in subcellular membrane localization of the substrates and protein-protein interactions (Aicart-Ramos et al., 2011; Zhang and Hang, 2017). The palmitoyl group is auto-acylated and enzymatically transferred to the protein by protein acyl transferases (Draper and Smith, 2009). The deacylation is catalyzed by acyl-protein thioesterases (APT1 and APT2) and PPT1. APTs are involved in the regulation of the reversible protein S-acylation. However, the role of PPT1, distinctly from other acyl-protein thioesterases, is considered only in the lysosomal degradation of the substrates (Won et al., 2018).

Defects in the PPT1 gene cause a severe infantile neurodegenerative lysosomal storage disorder (Vesa et al., 1995; Das et al., 2001; Simonati et al., 2009). The hallmarks of the disease are progressive neurodegeneration, blindness, seizures, cognitive decline, and shortened life span (Nita et al., 2016). Missense mutations are closely associated with later onset or a more extended disease course (Kousi et al., 2012). PPT1 is required for the morphological development and synaptic function of neurons (Koster and Yoshii, 2019). Moreover, the PPT1 deficient-mouse model displayed motor dysfunction at 3 months of age (Macauley et al., 2009). Although PPT1 plays important roles in developmental biology, the mechanistic understanding of PPT1 in muscle differentiation has not been explored.

Autophagy is a conserved self-degrading process that regulates the balance between the assembly of cellular components and the degradation of damaged cellular constituents (Wu and Lipinski, 2019). Lysosomal enzymes are involved in autophagic degradation and PPT1 depalmitoylates, the palmitoylated protein in lysosome, prior to their degradation (Koster and Yoshii, 2019). PPT1 is one of the target proteins upregulated by transcriptional factor EB (TFEB), the master controller of autophagy and lysosome biogenesis that influence the promoter region of coordinated lysosomal expression and regulation elements (Sardiello et al., 2009; Settembre et al., 2011, 2013). TFEB is also regulated by the activation of mammalian target of rapamycin complex 1 (mTORC1), which is a well-established regulator of the developmental process by coordinating cell growth and metabolism (Ge and Chen, 2012; Saxton and Sabatini, 2017a). Under nutrient-rich conditions, mTORC1 interacts with TFEB, and induces TFEB phosphorylation at S211 to inhibit autophagy (Napolitano and Ballabio, 2016). Conversely, under starvation conditions, TFEB is dissociated from mTORC1, and then translocated from the cytosol to the nucleus to activate lysosome biogenesis and autophagy (Roczniak-Ferguson et al., 2012). However, how autophagy is controlled by TFEB, mTORC1, and PPT1 during myoblast differentiation has not been explored.

Reactive oxygen species (ROS) are usually composed of superoxide (O_2_^−^), hydroxyl radicals (OH), and hydrogen peroxide (H_2_O_2_). It is well-known that ROS have been implicated as essential signaling molecules for metabolic processes including cellular proliferation, migration, apoptosis, and autophagy (Covarrubias et al., 2008; Zhang et al., 2016). In particular, increased ROS at physiological levels promote the adaptation of antioxidant responses to oxidative conditions during muscle differentiation. Moreover, mitochondrial ROS (mtROS) are produced as byproducts of the electron transport chain or by the dysregulation of cellular redox signaling (Zhang et al., 2016). Previous studies have reported that mitochondrial H_2_O_2_ plays a positive role during adipocyte, keratinocyte, stem cell, and muscle differentiation (Lee et al., 2011; Tormos et al., 2011; Hamanaka et al., 2013; Khacho et al., 2016). In addition, previous research showed that H_2_O_2_ activates nuclear factor erythroid 2-regulated factor 2 (Nrf2) during muscle differentiation (Al-Sawaf et al., 2014; Rajasekaran et al., 2020).

In this study, we evaluated whether mtROS regulate the induction of PPT1 which is an essential molecule of mTORC1-TFEB signaling pathway, required for myogenic autophagy during muscle differentiation and regeneration.

## Materials and Methods

### Cell Culture

C2C12 cells were purchased from ATCC (Manassas, VA, United States). C2C12 was maintained in Dulbecco modified Eagle’s medium (DMEM) containing 10% (v/v) fetal bovine serum (FBS), 100 U/ml penicillin, and 100 μg/ml streptomycin (proliferation medium, PM). Cells were induced to differentiate by replacing them in DMEM containing 2% (v/v) horse serum, 100 U/ml penicillin, and 100 μg/ml streptomycin (differentiation medium, DM). Full differentiation was achieved 5 days after induction. The differentiation was increased based on the morphology. The culture media were replaced with fresh media daily.

### Reagents and Antibodies

N-acetyl L-cysteine, 4,5-dihydroxy-1,3-benzenedisulfonic acid, and bafilomycin A1 were purchased from Sigma-Aldrich (St. Louis, MO, United States). Specific antibodies for myosin heavy chain (MHC), Nrf2, p62, lamin B, and β-actin were purchased from Santa Cruz Biotechnology (Dallas, TX, United States). Anti-SOD1 and superoxide dismutase 2 (SOD2) antibodies were purchased from AbFrontier (Seoul, Republic of Korea). The specific antibody for PPT1 was purchased from Sigma-Aldrich. Specific antibodies for lysosomal-associated membrane protein 1 (LAMP1), LAMP2, TFEB, phospho-TFEB (S211), mTOR, phospho-mTOR (S2448), p70S6K, phospho-p70S6K (S371 and T421/424), Atg3, Atg5, Atg7, Atg12, and LC3 were purchased from Cell Signaling Technology (Inc., Beverly, MA, United States).

### Flow Cytometry Analysis

Total intracellular ROS levels were measured with CM-H2DCFDA (DCF-DA) using a flow cytometer (Beckman Coulter, Inc., CA, United States). C2C12 cells were incubated with 5 μM DCF-DA at 37°C for 30 min. The mean DCF-DA fluorescence intensity was measured with excitation at 488 nm and emission at 525 nm. Mitochondrial superoxide levels were measured with MitoSOX using a flow cytometer (Beckman Coulter, Inc., CA, United States). C2C12 cells were incubated with 1 μM MitoSOX at 37°C for 30 min. The mean MitoSOX fluorescence intensity was measured with excitation at 510 nm and emission at 580 nm.

### PPT1 Enzyme Activity Assay

PPT1 enzymatic activity was measured as previously reported (Van Diggelen et al., 1999; Thada et al., 2016). C2C12 cells were collected and sonicated in double distilled water supplemented with protease inhibitor. Around 10 μg of total protein was incubated in a mixture containing 0.64 mM of substrate, 4-methylumbelliferyl-6-thio-Palmitate-b-D-glucopyranoside (CAYMAN CHEMICAL, Ann Arbor, MI, United States), 15 mM dithiothreitol, 0.375% (w/v) Triton X-100, and 0.1 U β-glucosidase from almonds (Sigma-Aldrich) in McIlvains phosphate/citric-acid buffer (4.0 pH). The total protein reaction mixture was then incubated for 1 h at 37°C. The reaction was stopped with the addition of 0.5 M NaHCO3/0.5 M Na2CO3 buffer (10.7 pH) with 0.025% Triton X-100, and fluorescence was measured by microplate reader (Bio-Rad, CA, United States). Relative enzymatic activity was estimated using the total fluorescence minus background.

### Reverse Transcription Polymerase Chain Reaction

For analysis of PPT1 messenger RNA (mRNA), total RNA was prepared using Trizol reagent (Thermo Fisher Scientific, Waltham, MA, United States) according to the manufacturer’s protocol. In short, C2C12 cells were lysed with 1 ml of Trizolreagent and were collected, mixed with 0.1 ml of chloroform, and centrifuged in a refrigerated centrifuge at 14,000 rpm for 15 min. The clear supernatants were separately collected, and the nucleic acids were precipitated using isopropanol. The precipitated total RNAs were washed by 70% ethanol, and then the pellets were dissolved by nuclease free water (Thermo Fisher Scientific, Waltham, MA, United States). The quantity and purity of RNA were estimated using a SynergyHTX Multi-Mode Microplate Reader equipped with a Take3 Micro-Volume Plate (BioTek Instruments, Inc., Winooski, VT, United States). Samples with optical density 260/280 value above 1.8 were used for further experiments. The complementary DNA (cDNA) was generated from the total RNA (1 μg) using the PrimeScript™ first strand cDNA Synthesis Kit (Takara Bio Inc., Shiga, Japan) according to the manufacturer’s protocol. The PCR products were amplified using PerfectShot™ Ex Taq (Takara Bio Inc., Shiga, Japan) and specific primers as follows: PPT1 forward, 5'-CCTGATCTCAGTTGGAGGAC-3'; PPT1 reverse, 5'-GGGCAGTAAACCATTCT-3'; GAPDH forward, 5'-CAACTTTGGCATTGTGGAAGGG-3'; and GAPDH reverse 5'-ACACATTGGGGGTAGGAACA-3'. The PCR reactions were conducted for 5 min at 95°C, followed by 25 cycles of 95°C for 30 s, 58°C for 30 s, and 72°C for 1 min. The amplified products were visualized on 1% agarose gel. GAPDH was used as a loading control.

### Promoter Analysis and Luciferase Assay

Serial Cloner^1^ (Version 2.6.1) was used to analyze the PPT1 promoter sequence and determine the DNA binding consensus of Nrf2. The 1,425-base pair (bp) of PPT1 promoter region and the antioxidant response element (ARE)-mutated fragment was chemically synthesized and cloned into the pGL3 plasmid vector by using XhoI and HindIII restriction enzymes (Cosmogenetech, Seoul, Republic of Korea). The pGL3 basic vector was purchased from Promega. C2C12 cells were transfected with 0.2 μg of pGL3 basic vector, pGL3-PPT1/Wt or pGL3-PPT1/Mt. along with the internal control plasmid, and pSV-β-Galactosidase (β-gal) Control Vector (Promega, Madison, WI, United States) with or without pcDNA3.0 or pcDNA3.0-Nrf2. After transfection, cells were incubated in PM or DM for 48 h. Luciferase assay was performed using Luciferase Assay System kit (Promega, Madison, WI, United States). The transfectants were lysed by lysis buffer that manufacturer provided. Luciferase and β-gal activities were measured in 50 μl cell lysates using a microplate reader (BioTek, Winooski, VT, United States), and the luciferase activity was normalized to the β-gal activity.

### Chromatin Immunoprecipitation Assay

A conventional chromatin immunoprecipitation (ChIP) assay was conducted as described previously (Kim et al., 2008). C2C12 cells were grown under PM and DM conditions, and then fixed by addition of formaldehyde to the media to final concentration of 1%. To harvest cells, plates were rinsed with cold phosphate buffered saline (PBS), covered with 10 ml of 5% FBS in PBS, and then scraped. Chromatin was prepared using SimpleChIP® Enzymatic Chromatin IP Kit (Cell Signaling Technology, Danvers, MA, United States) according to the manufacturer’s protocol, with 25 s sonication pulses at 10 s intervals, which yielded chromatin fragments of an apparent size of 100–400 bp. An aliquot from each sample representing 2% of the total volume was used as the input fraction and was processed with the eluted immunoprecipitates beginning at the cross-link reversal step. Equal amounts of chromatin from each sample were incubated at 4°C overnight with 5 μl of antibodies against Nrf2. The PPT1 promoters containing the Nrf2 binding sites were amplified using the following primers: candidate for −3,309 bp forward, 5'-CCAGCAAAACCCAGGGA-3' (start site: −3,209 bp); candidate for −3,309 bp reverse, 5'-AAATCAGAGTAAAAACAAAGGAAA G-3' (start site: −3,402 bp) and candidate for −1,269 bp forward, 5'-TCATTGTTAACTTCATAACTGTA-3' (start site: −1,065 bp); and candidate for −1,269 bp reverse, 5'-TAAGTCTTTGGCCTACCTTT-3' (start site: −1,369 bp). The PCR reactions were conducted for 5 min at 95°C, followed by 40 cycles of 95°C for 30 s, 55°C for 30 s, and 72°C for 30 s. The amplified products were visualized on 2% agarose gel. The PCR product sizes are 193 and 214 bp, respectively.

The precipitated chromatins were also quantified by real-time PCR (RT-PCR) with same primers using SYBR® Green PCR Master Mix and the Applied Biosystems™ 7500 Real-Time PCR system (Applied Biosystems, Foster City, CA, United States) according to the manufacturer’s instructions. The samples were first denatured at 95°C for 15 min, followed by 40 cycles of denaturation at 95°C for 15 s, annealing at 60°C for 15 s, and elongation at 72°C for 20 s. Finally, the samples were held at 65°C, and melting curves were conducted from 65 to 95.1°C. All tests were performed in triplicate, and all experiments were repeated three times. Nonspecific antibody (IgG) served as a negative control. The relative levels of the fragments of interest in the immunoprecipitated DNA were determined from the threshold cycle (Cᴛ) for each PCR reaction. The percentage of input was calculated: ΔCᴛ _normalized ChIP_ = Cᴛ_ChIP_−[Cᴛ_Input_ − Log2^(Input Dilution Factor)^], Input Dilution Factor = (fraction of the input chromatin)^−1^ = (2%)^−1^=50; %Input = 2^−ΔCᴛ normalized ChIP^ × 100%.

### Small Interference RNA

Palmitoyl protein thioesterase 1 small interference RNAs (siRNAs; #1 sense, 5'-GAUACAAUGCUAUUGGCUU-3'; #1 antisense, 5'-AAGCCAAUAGCAUUGUAUC-3'; #2 sense, 5'-GUUCUCACAUCUGCGACUU-3'; and #2 antisense, 5'-AAGUCGCAGAUGUGAGAAC-3') and control-siRNA (sense, 5'-UCCCAGAUAGAGACUUCA-3'; antisense, 5'-UUGAAGUCUCUAUCUGGGATT-3') were purchased from Sigma-Aldrich. The siRNAs (100 nM/60 mm dish) were transfected into C2C12 cells by using TransIT®-2020 Transfection Reagent (Mirus Corp., Madison, WI, United States) according to the manufacturer’s instructions. When cells reached 50–60% confluence, they were transfected with each siRNAs, and further incubated for 24 h. The efficiency of siRNAs interference of PPT1 was monitored by western blot analysis.

### Confocal Microscopy Observations

For LC3 puncta determination, C2C12 cells were plated onto glass coverslips. After 5 days of differentiation in DM, the cells were incubated with 1 μM of LysoTracker™ Red DND-99 and Lysosensor Green DND-189 (Thermo Fisher Scientific, Waltham, MA, United States) for 30 min, and then fixed with 4% paraformaldehyde for 30 min and rinsed with PBS twice. The coverslips were then permeabilized and blocked with 5% bovine serum albumin in PBS for 1 h at room temperature, and were incubated with anti-rabbit mTORC1 and LC3 primary antibodies (1:200, Cell Signaling Technology, Inc., Beverly, MA, United States) overnight at 4°C, followed by secondary antibody (1:300, FITC-conjugated goat anti-rabbit IgG, ThermoFisher Scientific, Waltham, MA, United States). Nuclei were stained with 4′,6-diamidino-2-phenylindole (DAPI), rinsed, and mounted in Vectashield mounting medium (Vecta Laboratories, Inc., Burlingame, CA, United States). Images were acquired using a Zeiss LSM800 confocal laser scanning microscope.

### Preparation of Nuclear Extracts

Nuclear extracts were obtained using NE-PER nuclear and cytoplasmic extraction kit from ThermoFisher (Cat #78835). Confluent cells were detached by trypsinization and centrifuged at 90 × *g* at 4°C for 5 min. Cells were resuspended in PBS, centrifuged at 20,000 × *g* at 4°C for 5 min, and once again resuspended in 200 μl of buffer (10 mM HEPES, pH7.9, 10 mM KCl, 1 mM DTT, 0.5 mM PMSF, and 0.1 mM EDTA). After incubation on ice for 10 min, cells were lysed by addition of 12.5 μl of 10% NP-40. Harvesting of the nuclei was accomplished by centrifugation at 20,000 × *g* at 4°C for 2 min. The supernatant (cytosolic extract) was transferred to a new tube, and the nuclear pellets were resuspended in 50 μl of extraction buffer (20 mM HEPES, pH 7.9, 0.4 M NaCl, 1 mM DTT, 1 mM PMSF, 1 mM EDTA, and 1% NP-40) and incubated on ice for 10 min. Nuclear debris were removed by centrifugation at 20,000 × *g* at 4°C for 10 min, and the supernatant was used as nuclear protein extract.

### Cathepsin Enzyme Activity Assay

The enzymatic activities of cathepsin B, D, and K were measured using a fluorometric assay kit from Abcam (ab65300, ab65302, and ab65303, respectively), according to the manufacturer’s instructions. Briefly, C2C12 cells were washed twice in ice-cold PBS and then homogenized in extraction buffer, as described by the manufacturer. After incubation on ice, the extract was centrifuged at 10,000 × *g* at 4°C for 5 min, and 50 μl of supernatant was mixed with an equal volume of 2 × reaction buffer and 2 μl of substrate in a 96-well microplate. Plates were kept in the dark at 37°C for 1 h, and fluorescence was recorded using a microplate reader (Bio-Rad, CA, United States). Protein concentration was determined by the Bradford assay (Bio-Rad, CA, United States). Cathepsin B, D, and K activities were determined by fluorometry at Ex/Em = 400/505, 328/460, and 400/505 nm, respectively.

### Muscle injury

The left mouse tibialis anterior (TA) muscle was injured by a cardiotoxin (CTX) injection. Before the procedure, the mice were anesthetized. TA muscles were injected with 50 μl of 10 mM CTX solution in 0.9% NaCl. CTX-injured muscles were dissected on 1, 2, 4, 6, and 8 days following injury. Immediately after isolation, the muscles were frozen in liquid nitrogen and preserved at −80°C. These tissues were homogenized and lysed with lysis buffer (50 mM Tris-HCl, pH 7.4, 0.1% NP40, and 150 mM NaCl) containing protease and phosphatase inhibitors and assessed by western blot analysis. In every experiment, at least three animals were analyzed for each time point after CTX injury.

### Western Blot Analysis

Cells were lysed with radioimmunoprecipitation assay (RIPA) buffer (50mM Tris-HCl, pH 7.4, 150mM NaCl, 5mM EDTA, 1% Na-deoxycholate, and 1% NP-40), supplemented with protease inhibitors (200mM PMSF, 200mM Na_2_VO_4_, and 200mM NaF). The TA muscles were lysed with protein extraction solution (Intron Biotechnology, Chinju, Korea). Protein concentrations of lysates were measured using a Bio-Rad DC protein assay (Bio-Rad Laboratories, Inc., Hercules, CA, United States). The lysates were separated by 8 ~ 12% sodium dodecyl sulfate polyacrylamide gel electrophoresis (SDS-PAGE) and transferred onto a nitrocellulose membrane (Pall Corporation, Washington, NY, United States) for 1.5 h at 100 V at 4°C. The membranes were blocked with 3% bovine serum albumin in TBST (20 mmol/L Tris-HCl, pH 7.5, 50 mmol/L NaCl, and 0.1% Tween 20). After blocking, the membranes were incubated for 1 h with primary antibodies diluted to 1:1,000 in TBST. After washing, the membranes were incubated for 1 h with horseradish peroxidase-labeled secondary antibody diluted to 1:2,000 ~ 5,000 in TBST. The protein bands were then visualized by an enhanced chemiluminescence kit (Santa Cruz Biotechnology, Dallas, TX, United States) and detected with an AI600 Imager (GE Healthcare, Chicago IL, United States). Quantitative results are presented as the ratio of the band intensity of the protein of interest to the band intensity of β-actin.

### Statistical Analysis

The results are expressed as mean ± SE of the mean of at least three independent experiments. The differences between two means were analyzed with the Student’s *t*-test and were considered statistically significant when the *p* values were < 0.05.

## Results

### PPT1 Is Increased During Muscle Differentiation

**Figure 1:**
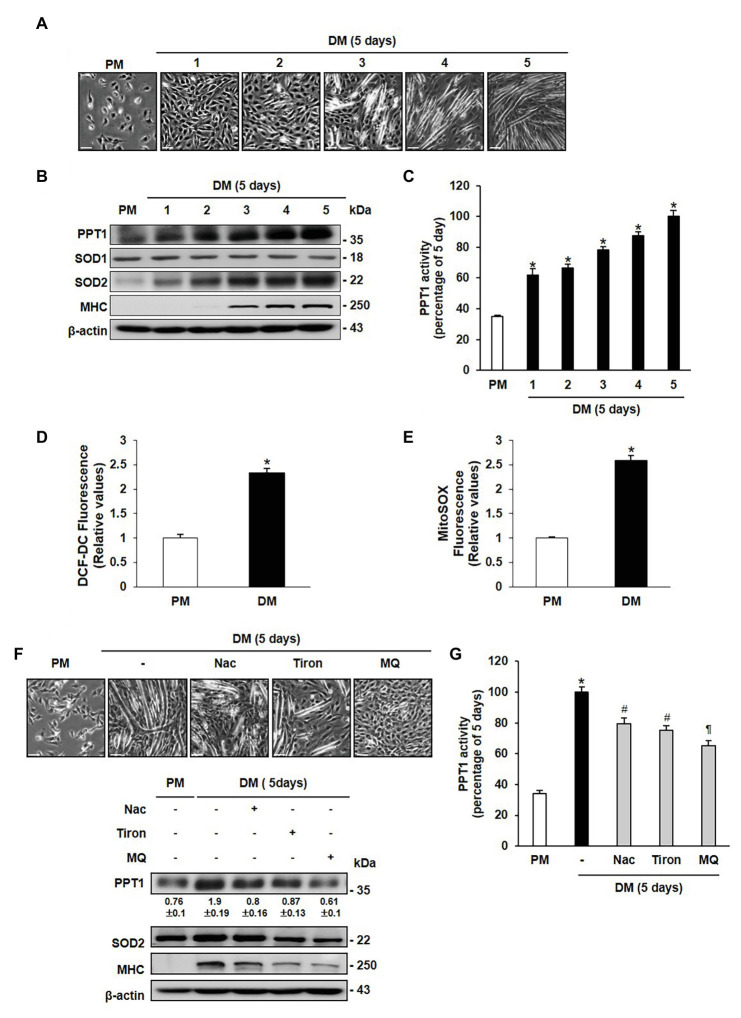
Palmitoyl protein thioesterase 1 (PPT1) is increased during muscle differentiation. **(A)** Morphological changes were observed at 5-day after induction of differentiation in C2C12 cells. **(B)** Expressions of PPT1, myosin heavy chain (MHC), SOD1, and superoxide dismutase 2 (SOD2) were analyzed during muscle differentiation by western blot analysis. β-Actin is used as loading control. **(C)** Activity of PPT1 was determined by fluorometry at 358 and 448 nm excitation and emission wavelengths during muscle differentiation, and expressed by percentage.^*^p < 0.01, compared to cells in proliferation medium (PM). **(D)** Reactive oxygen species (ROS) levels were detected by DCF-DA after 24 h of differentiation medium (DM) using FACS analysis.^*^p < 0.01, compared to cells in PM. **(E)** Mitochondrial ROS (mtROS) levels were analyzed by Mitosox fluorescence after 24 h of DM. ^*^p < 0.01, compared to cells in PM. **(F)** Cells were treated with 2 mM NAC, 250 μM Tiron, and 250 μM MitoQ 5 days after induction of differentiation, and observed morphological changes and expression levels. **(G)** PPT1 activity was measured after treatment with antioxidant for 5-day differentiation by fluorometry at 358 and 448 nm excitation and emission wavelengths, and expressed by percentage. ^*^p < 0.01, compared to cells in PM. ^#^*p* < 0.05, compared to cells in DM. *p* < 0.01, compared to cells in DM. In graphs, the data shown represent the mean ± SE of three independent experiments. Scale bar: 500 μm.

To investigate the role of PPT1 during muscle differentiation, we examined the morphological changes in C2C12 cells. Replacement of PM with DM results in the formation of multinucleated myotubes in a day-dependent manner (Figure 1A). The expression level of MHC steadily increased until 5 days (Figure 1B). PPT1 expression and activity also gradually increased during muscle differentiation (Figures 1B,C). We next tested whether ROS levels were elevated during muscle differentiation. SOD2 expression level was remarkably increased without changes of SOD1 expression (Figure 1B; Kuwahara et al., 2010; Lustgarten et al., 2011; Gordon et al., 2019). Moreover, cytosolic and mitochondrial ROS levels were significantly elevated for 24 h in DM compared to PM (Figures 1D,E).

To further examine whether expression of PPT1 is induced by cytosolic and mitochondrial ROS, we treated cells with NAC (a glutathione mimic), Tiron (a superoxide scavenger), and Mitoquinone (MitoQ, a mitochondria-targeted antioxidant) in culture medium. Treatment with these antioxidants resulted in suppression of PPT1 expression and activity, as well as decrease in myotube formation and MHC expression levels (Figures 1F,G). These outcomes show that PPT1 was increased and was inhibited by antioxidant during muscle differentiation.

### PPT1 Promoter Is Transactivated by Nrf2 During Muscle Differentiation

**Figure 2:**
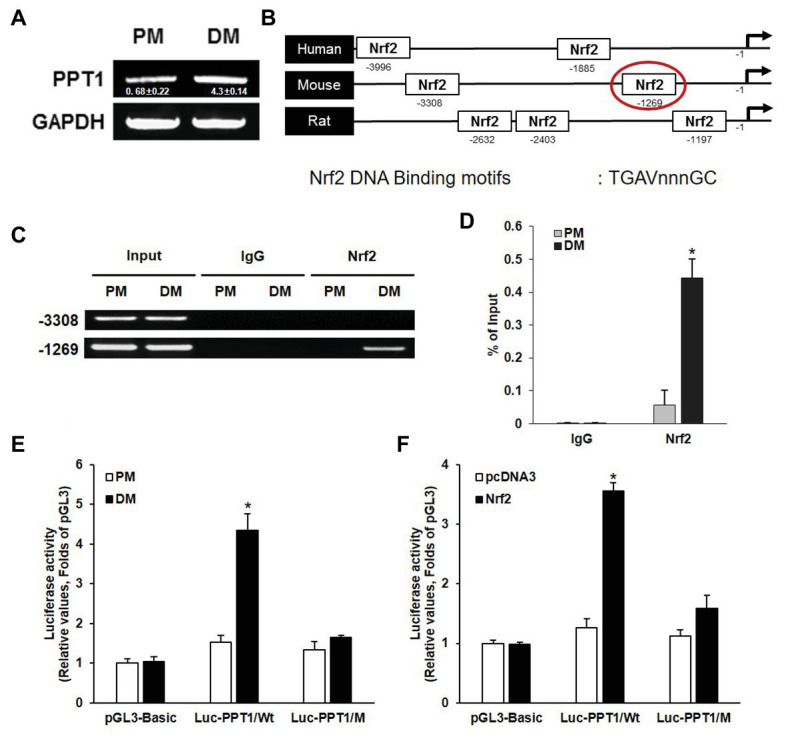
PPT1 is transcriptionally upregulated by nuclear factor erythroid 2-regulated factor 2 (Nrf2) during muscle differentiation. **(A)** messenger RNA (mRNA) expression of PPT1 was observed *via* real-time PCR (RT-PCR) at 5-day after induction of differentiation. GAPDH is used as loading control. **(B)** Map of putative Nrf2 binding sites in PPT1 promoter region. **(C)** Chromatin from individual samples was precipitated using antibodies against Nrf2 and control IgG in chromatin immunoprecipitation (ChIP) assay for 5-day differentiation. Input genomic DNA was used as a positive control, and nonspecific IgG was used as a negative control. **(D)** ChIP assay at −1,269 region were observed *via* RT-PCR in PM and DM. ^*^p < 0.01, compared to cells in PM. **(E)** PPT1 promoter activities were monitored. Cells were transiently transfected with the pGL3 empty vector in PM and DM.^*^p < 0.01, compared to cells in PM. **(F)** Cells transfected with pcDNA3-Nrf2 were observed in PM and DM. ^*^p < 0.01, compared to cells in PM.

Previous studies have demonstrated that ROS activate Nrf2 and induce muscle differentiation (Piao et al., 2005; De Vries et al., 2008). Thus, to investigate whether the expression of PPT1 is transcriptionally upregulated during muscle differentiation, we first evaluated the expression level of PPT1 mRNA during muscle differentiation. As expected, the expression of PPT1 mRNA was markedly increased in 5 days of muscle differentiation (Figure 2A). Next, we analyzed the promoter region of the PPT1 gene by 5 kb and identified two putative Nrf2 binding sequences, 5′-TGAVNNNGC-3′, which were located at −3,308 and −1,269 bp upstream of PPT1 ORF (Figure 2B). To investigate the binding site of Nrf2 to the PPT1 promoter region, we conducted ChIP-assay for muscle differentiation. Our results showed that the binding sequence at −1,269 bp upstream of the PPT1 ORF was associated with Nrf2 (Figures 2C,D) using Electrophoresis and Real-Time PCR.

To further confirm the response of the ARE sequence located at −1,269 bp in the muscle differentiation condition, we designed two luciferase reporter constructs, wild type (pGL3-PPT1), and mutated ARE type (pGL3-PPT1/M) and conducted the luciferase assay. The pGL3-PPT1-transfected cells showed significant transactivation under DM conditions, while pGL3-PPT1/M-transfected cells, which contained 5′-AAAAGCAGC-3′ instead of the wild-type motif at the ARE site, did not exhibit significant changes under DM conditions (Figure 2E). In addition to, the activity of Nrf2 transactivation on the PPT1 promoter, we overexpressed Nrf2 *via* transfection of the pcDNA3-Nrf2 expression vector under PM conditions. The results were similar to those observed under DM conditions, in terms of luciferase activity (Figure 2F). Additionally, PPT1 knockdown-cell promoted levels of ROS and Nrf2 translocation to nucleus (Supplementary Figure 1). These results indicate that the expression of PPT1 is transcriptionally induced by Nrf2 during muscle differentiation.

### Inhibition of PPT1 Attenuates the Muscle Differentiation

**Figure 3:**
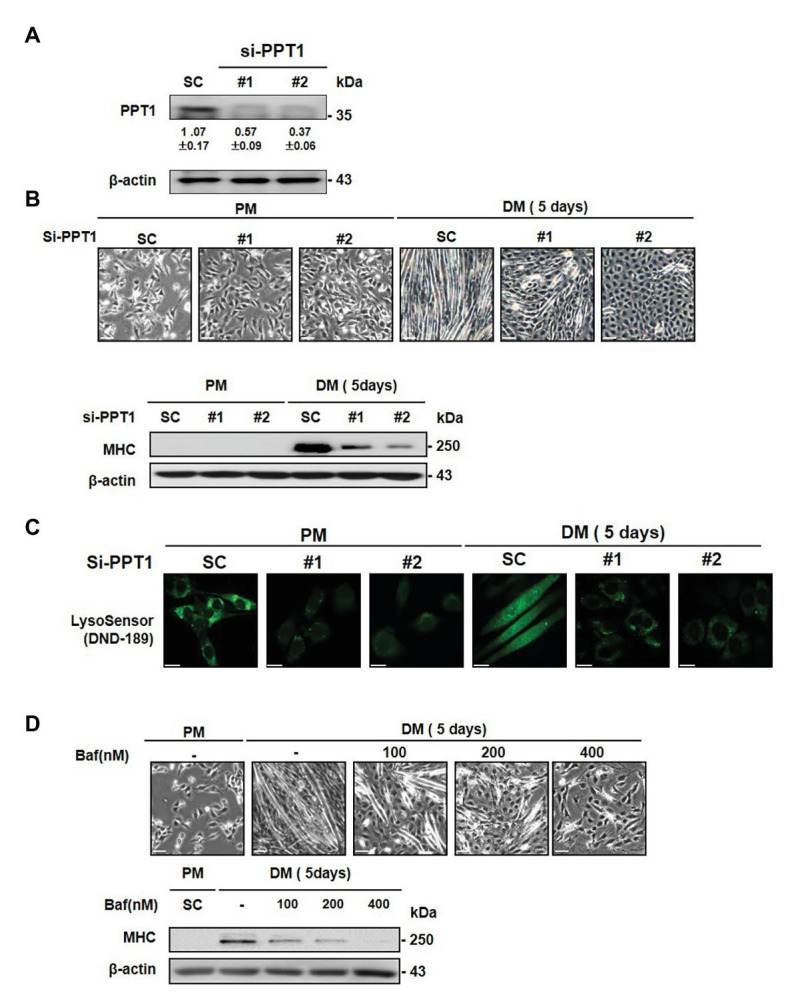
Inhibition of PPT1 attenuates muscle differentiation. **(A)** C2C12 cells were transfected with 100 nM two different PPT1 small interference RNAs (siRNAs; si#1 and si#2) for 48 h, and then PPT1 expressions were assessed by western blot. **(B)** Cells were further incubated in DM for 5-day after transfected with 100 μM siRNAs, and morphological changes were observed. The expression of MHC was evaluated by western blot analysis for 5-day differentiation. **(C)** Fluorescence was visualized by confocal microscopy by pH-sensitive LysoSensor DND-189 in PM and DM. **(D)** Cells were treated with bafilomycin A1 at 125, 250, and 500 nM in DM for 5 days. Cell picture scale bar: 500 μm and confocal imaging scale bar: 200 μm.

To further explore the roles of PPT1 in muscle differentiation, we employed commercially available siRNAs. PPT1 knockdown blocked morphological changes in myofiber formation and MHC expression, compared to scrambled siRNA-transfected cells (SC; Figures 3A,B). Previous studies have demonstrated that PPT1 knockout cells increase lysosomal pH though suppression of v-ATPase V0a1 activity (Bagh et al., 2017; Rebecca et al., 2017, 2019). Thus, we next examined whether knockdown of PPT1 causes changes in lysosomal pH during muscle differentiation. Through confocal microscopy analysis and the use of DND-189, we showed that PPT1-knockdown cells present a markedly increased lysosomal pH compared to SC in PM and DM conditions (Figure 3C). Consistent with this, we confirmed the pharmacological inhibitor bafilomycin A1, which suppresses v-ATPase activity (Sandri, 2010). Treatment with bafilomycin A1 suppressed the formation of morphological changes and expression of MHC (Figure 3D). Additionally, treatment of bafilomycin A1 increased lysosomal pH compared to PM and DM conditions (Supplementary Figure 2). These findings indicated that the defect in PPT1 leads to increased lysosomal pH and blocks muscle differentiation.

### PPT1 Is an Essential Molecule for Maintaining Lysosomes

**Figure 4:**
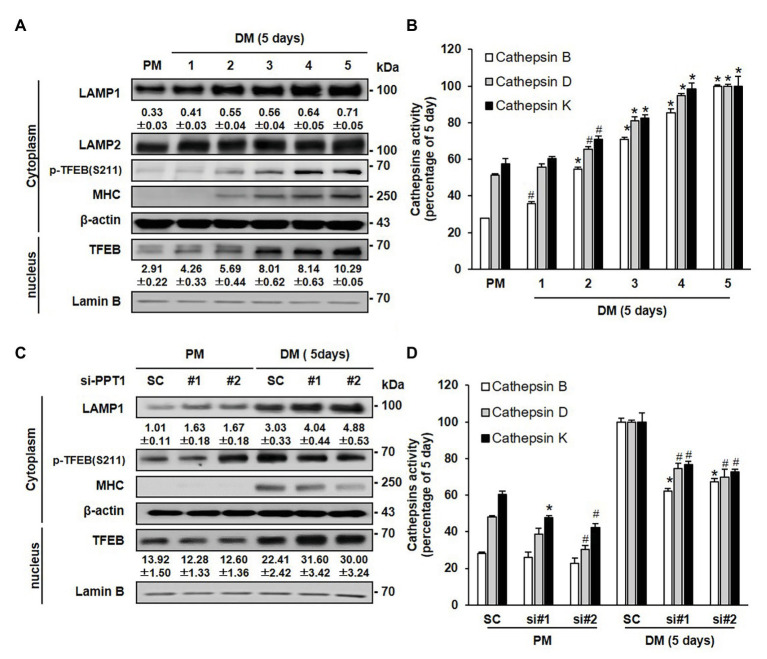
PPT1 is an essential molecule maintaining lysosomes. **(A)** The expression levels of lysosomal-associated membrane protein 1 (LAMP1), LAMP2, transcriptional factor EB (TFEB), phospho-TFEB at serine 211 (S211), and MHC on the indicated 5-days were evaluated by western blot analysis. Lamin-B is used as nuclear protein loading control. **(B)** Cathepsin B, D, and K activity was determined during myoblast differentiation by fluorometry at Ex/Em = 400/505 nm, 328/460 nm, and 400/505 nm then expressed by percentage, ^#^*p* < 0.05, compared to cells in PM. ^*^p < 0.01, compared to cells in PM. **(C)** C2C12 cells transfected with PPT1 siRNAs were incubated in DM for 5-days, and were assessed by western blot analysis. **(D)** Under the same conditions as in **(C)**, cathepsin activities were measured during 5-day differentiation by fluorometry and expressed by percentage, ^#^*p* < 0.05, compared to cells in scramble siRNA. ^*^p < 0.01, compared to cells in scramble siRNA.

To further investigate the lysosomal changes induced by the suppression of PPT1 during muscle differentiation, we examined the levels of lysosomal proteins during muscle differentiation. The expression of LAMP1 protein, a lysosome marker, gradually increased during muscle differentiation without changes in LAMP2 (Figure 4A). Phosphorylation of TFEB, a well-known master controller of lysosomal biogenesis (Vega-Rubin-De-Celis et al., 2017), was also elevated and translocated from the cytosol to the nucleus during muscle differentiation (Figure 4A). In addition, as number of increased lysosome, we investigated activity of Cathepsin B, D, and K, well-known represented lysosomal hydrolase (Kirschke et al., 1983; Colella et al., 1986; Bechet et al., 1991; Ebisui et al., 1994; Ogasawara et al., 2018). Cathepsin B, D, and K activities were elevated during muscle differentiation (Figure 4B). In previous results, PPT1 knockdown-cell showed increased lysosomal pH. Therefore, we investigated whether inhibition of PPT1 causes the changes of lysosomal protein, PPT1 knockdown-cells had an enhanced expression of LAMP1 and TFEB when compared to SC under DM conditions (Figure 4C). However, the activity of cathepsin proteins in PPT1 knockdown cells was significantly decreased compared to that in SC under DM conditions (Figure 4D). These results indicate that the dysfunction of the lysosome by knockdowns of PPT1 induces the upregulation of non-functional lysosomal proteins.

### Deficiency of PPT1 Blocks mTORC1 Activation

**Figure 5:**
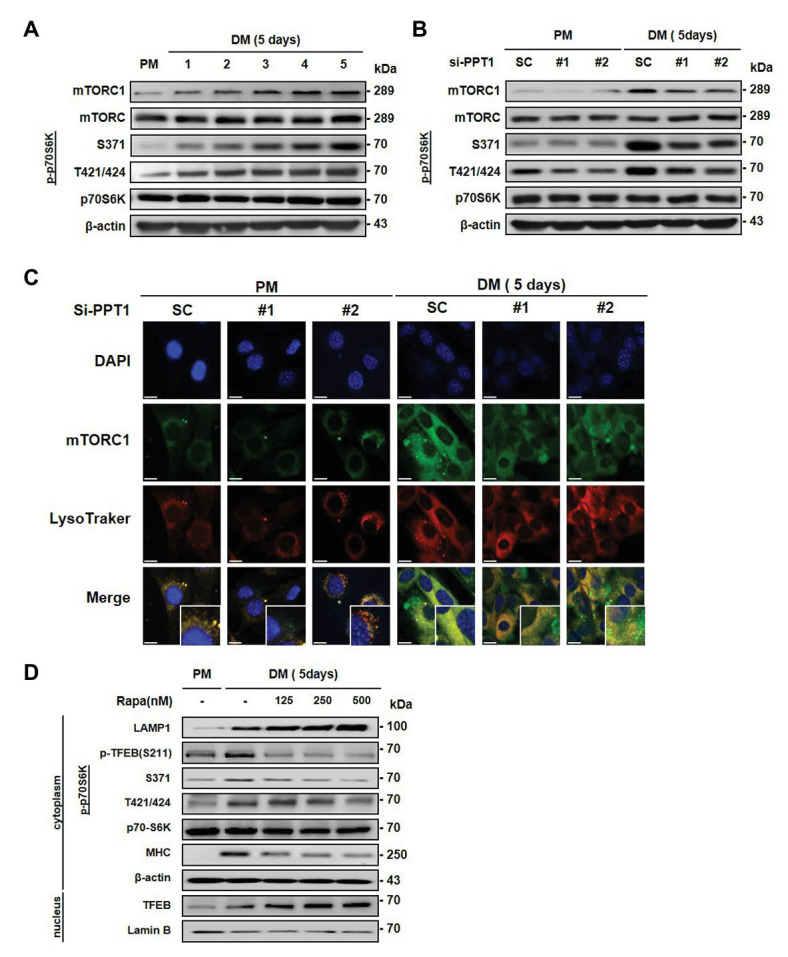
Deficiency of PPT1 blocks mammalian target of rapamycin complex 1 (mTORC1) activation. **(A)** The levels of mTOR, phospho-mTOR serine 2448 (S2448), p70S6K, phospho-p70S6K at serine 371 (S371), and threonine 421/serine424 (T421/S424) were analyzed during myoblast differentiation by western blot analysis. **(B)** C2C12 cells transfected with PPT1 siRNAs were incubated in DM for 5 days, and then expression of mTOR, p-mTOR, p70S6K, and p-p70S6K was assessed by western blot analysis. **(C)** mTORC1 inactivation was observed by confocal microscopic analysis in 5-day differentiation. **(D)** Cells were treated with 125, 250, and 500 nM rapamycin for 5-day and analyzed by western blot analysis. Scale bar: 200 μm.

Previous studies have shown that mTORC1 is only activated in the presence of nutrients and growth factors, which induces its movement to the lysosome surface (Saxton and Sabatini, 2017b). In addition, mTORC1 is known to stimulate muscle differentiation (Yoon, 2017). Moreover, mTORC1 regulates phosphorylation of TFEB and inhibits its nuclear translocation (Kim and Guan, 2019). Consistent with this, we found that the expression of mTORC1 and phospho-p70S6K was gradually promoted during muscle differentiation (Figure 5A). Next, we tested whether the elevation of TFEB translocation by PPT1 knockdown affects the expression of mTORC1 during muscle differentiation. The silencing of PPT1 diminished the expression levels of mTORC1 and phospho-p70S6K in DM (Figure 5B). Interestingly, confocal microscopic analysis showed that reduced mTORC1 expression exhibited a limited translocation to the lysosome compared to SC in a 5-day differentiation process (Figure 5C). Consistently, treatment with rapamycin, an mTORC1 inhibitor, dramatically upregulated the expression levels of LAMP1 and translocation of TFEB from the cytosol to the nucleus compared to SC (Figure 5D). These outcomes show that PPT1 plays an essential role in mTORC1 activation.

### PPT1 Is Required for the Degradation of Autophagy Flux During Muscle Differentiation

**Figure 6:**
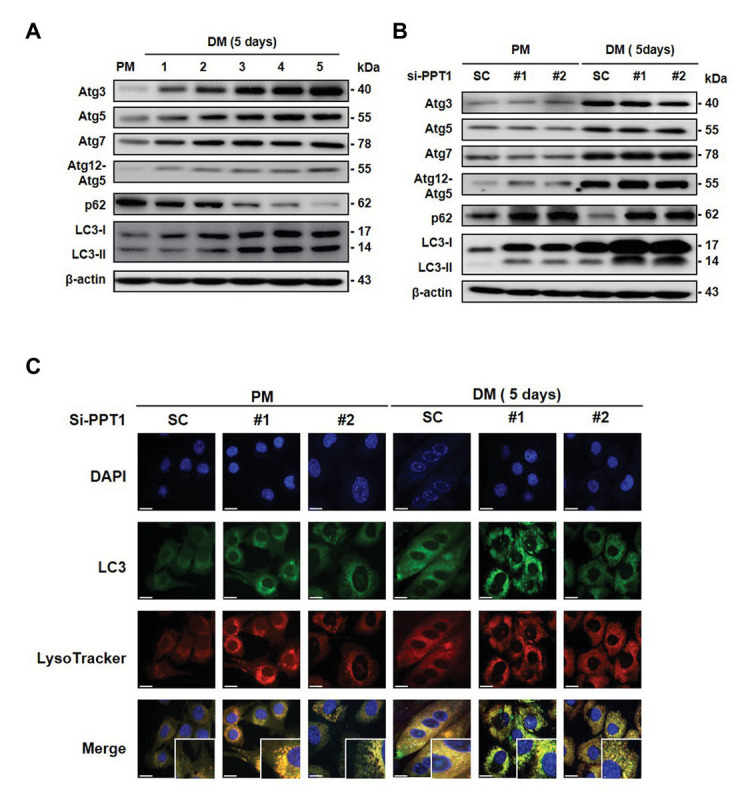
PPT1 is required for the degradation of autophagy flux during muscle differentiation. **(A)** Expression of Atg3, Atg5, Atg7, Atg12–5, p62, and LC-3 was accessed during muscle differentiation. **(B)** C2C12 cells were transfected with PPT1 siRNAs in DM, and then expression of autophagy-related gene (Atg) proteins, p62, and LC3-II conversion was evaluated in PM and DM by western blot analysis. **(C)** Under the same conditions as in **(B)**, fluorescence was visualized by confocal microscopic analysis. Scale bar: 200 μm.

It is well-established that autophagy is activated during muscle differentiation for muscle fiber formation (Ownby et al., 1993). Consistent with this, we found that the expression levels of autophagy-related gene (Atg) proteins and LC3 conversion gradually increased, and p62, which is the classical receptor of autophagy was degraded during muscle differentiation (Figure 6A). We next investigated whether silencing of PPT1 affects the change in Atg proteins and autophagic degradation. Although the knockdown of PPT1 did not change the Atg proteins, accumulation of LC3 and p62 was generated during muscle differentiation (Figure 6B). In addition, confocal microscopic analysis indicates that silencing of PPT1 compared to SC, promoted the accumulation of autophagy flux during muscle differentiation under both PM and DM conditions (Figure 6C). These findings indicated that the defect in PPT1 induced the accumulation of autophagy flux without changes in autophagosome formation.

### Transient Expression of PPT1 During Muscle Regeneration Following Acute Injury

**Figure 7:**
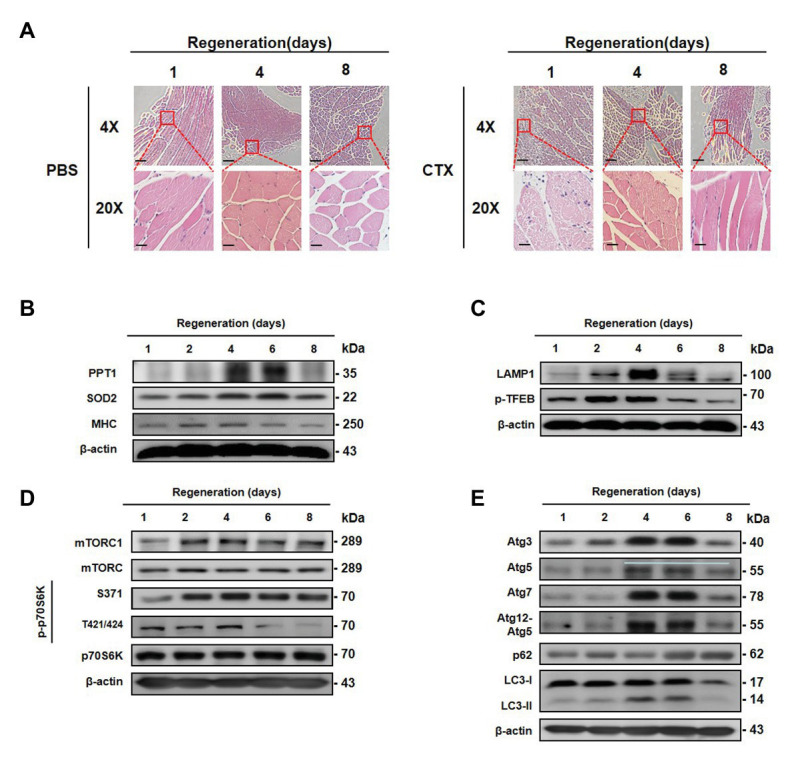
Transient expression of PPT1 during muscle regeneration following acute injury. **(A)** Mouse left tibialis anterior (TA) muscles were injected with 50 μl (10 mM) cardiotoxin (CTX). TA muscles were stained with hematoxylin-eosin. **(B)** Mouse TA muscle tissues were homogenized and assessed by western blot analysis. The expression levels of PPT1, SOD2, and MHC were elevated. **(C)** The expression levels of lysosome biogenesis marker including LAMP1 and phospho-TFEB at serine 211 (S211) were evaluated. **(D)** The expression levels of mTOR, phospho-mTOR serine 2448 (S2448), p70S6K, phospho-p70S6K at serine 371 (S371), and threonine 421/serine424 (T421/S424) were analyzed by western blot analysis. **(E)** The expression levels of Atg3, Atg5, Atg7, Atg12–5, p62, and LC-3 were accessed by western blot analysis. 4X scale bar: 500 μm and 20X scale bar: 100 μm.

To determine whether expression of PPT1 participates in muscle regeneration *in vivo*, we injected CTX, inducer of muscle necrosis (Azad et al., 2009), into the TA muscle, and mice are euthanized at 1, 2, 4, 6, and 8 day after injection. TA muscle damage increased up to 6 days after CTX injection, and recovery was observed on day 8 (Figure 7A). Expression of PPT1, SOD2, and MHC levels gradually increased during TA muscle regeneration (Figure 7B). LAMP1, p-TFEB, mTOR, mTORC1, and p70S6K were remarkably upregulated after CTX injection (Figures 7C,D). Autophagy markers were promoted during muscle regeneration (Figure 7E).

## Discussion

Although lysosomal storage disease (LSD) is a well-known manifestation of metabolic disorders that cause defects in lysosome function in neurons, kidneys, and melanomas, it has not yet been reported in muscle differentiation (Surendran et al., 2014; Bagh et al., 2017; Rebecca et al., 2019). Here, we report for the first time that PPT1 gene expression is upregulated *via* transcriptionally induced Nrf2 in muscle differentiation. Exogenous antioxidant treatment repressed PPT1 expression and, impaired muscle differentiation. We also found that PPT1 is interrelated with lysosomal function. Moreover, inhibition of PPT1 expression attenuated autophagic degradation. Intracellular ROS are known to be essential molecules for muscle differentiation (Lee et al., 2010). Previous studies have reported that an increase in mitochondrial H_2_O_2_, which was dismutated from mitochondrial O_2_^−^ by SOD2, is required to induce muscle differentiation (Kim et al., 2018). Furthermore, when ROS levels are increased up to pathological levels, the ARE pathway defends against cellular oxidative stress (Piao et al., 2005; De Vries et al., 2008). In the current study, we demonstrated through a bioinformatics analysis that the consensus Nrf2 binding sequences exist in the human, mouse, and rat PPT1 promoter. One of these, which was located at −1,269 bp upstream of the mouse PPT1, start codon ORF directly bound to Nrf2 during muscle differentiation. Thus, PPT1 is transcriptionally induced through Nrf2 activation during muscle differentiation.

To date, the characterization of PPT1 and TFEB signaling pathway has not been reported during muscle differentiation. Our findings showed that the defect of PPT1 with its specific siRNA attenuated the lysosome function and muscle differentiation. In addition, we previously reported that the inhibition of autophagosome formation and fusion with lysosomesby compounds such as bafilomycin, chloroquine, 3-Methyladenine, and wortmannin attenuated muscle differentiation (Kim et al., 2018). The lysosome is thought to be an essential organelle during cell development (Ballabio and Gieselmann, 2009; Shen and Mizushima, 2014; Perera and Zoncu, 2016). Furthermore, PPT1 knockdown attenuated the activity of cathepsin proteins unlike elevated TFEB and LAMP1, indicating that inhibition of PPT1 causes accumulation of non-functional lysosomes and lipidated proteins.

The myogenic controller mTOR is well-known not only for its roles in the development but also in the inactivation process of TFEB by serine phosphorylation (Vega-Rubin-De-Celis et al., 2017; Napolitano et al., 2018). Under sufficient nutrient conditions, mTORC1 is recruited to be activated for lysosomal surface positioning (Lawrence and Zoncu, 2019). Consistently, we found that mTORC1 was inactivated by inhibition of PPT1, and treatment with rapamycin diminished muscle differentiation. However, TFEB was translocated from the cytosol to the nucleus when mTORC1 is activated during muscle differentiation, indicating that both TFEB and mTORC1 are elevated to facilitate cellular remodeling during muscle differentiation. Previously, the relationship between PPT1 and mTORC1 was not known during muscle differentiation. Our findings suggest that suppression of PPT1 inhibits mTORC1 activation in nutrient-rich conditions. Autophagy is well-known to not only assist in cellular development but also inhibit the activation of mTORC1 (Mizushima and Levine, 2010; Nnah et al., 2019). Much of our understanding of mTOR is restricted in the context of suppressingeffects on autophagy in nutrient-enriched conditions. However, myoblast differentiation requires autophagy to assist the removal of pre-existing structures and elevate cellular remodeling, indicating that the autophagy mechanism during muscle differentiation is completely different. In addition, our results showed that PPT1 knockdown-cells were increased levels of ROS, Nrf2 translocation, and accumulation of p62. In previous studies, Nrf2 was upregulated by accumulation of p62, which competes with Nrf2 for Keap 1 binding. Our results showed that autophagy does not act as mTORC1 inactivator during muscle differentiation, and PPT1 is induced by Nrf2 in muscle differentiation but in pathological processes such as inhibition of PPT1, the accumulation of p62 seems to be increase Nrf2 compare to physiological process (Bellezza et al., 2020). In addition, our findings indicate that PPT1 regulates mTORC1 activation and TFEB phosphorylation, resulting in the molecule that controls autophagic degradation. However, suppression of PPT1 did not result in changes in autophagosome formation, indicating that autophagosome formation is an independent mechanism regardless of the inactivation of mTORC1 and TFEB in dysfunctional lysosomes. In addition, autophagosomes are not produced in the dysfunction of lysosomes, and only lysosomes are continuously formed, it is thought that it is regulated by specific signaling molecules. Therefore, it is important to further study molecules that recognize the dysfunction of lysosomes during myoblast differentiation. In addition to, skeletal muscle regeneration is a physiological process that occurs in skeletal muscle in response to disease or injury. CTX is well-known acute-injury-induced skeletal muscle regeneration inducer. Our findings showed that during muscle regeneration *in vivo*, expression levels of PPT1 and SOD2 were increased, and lysosome biogenesis, mTORC1, and autophagy were activated.

In conclusion, we demonstrated for the first time that elevated ROS levels induce the expression of PPT1.We also showed that PPT1 is transactivated *via* Nrf2 during muscle differentiation. Lastly, we demonstrated that PPT1 might play important roles in maintaining lysosomal degradation by the mTORC1-TFEB signaling pathway during myoblast differentiation.

## Data Availability Statement

The raw data supporting the conclusions of this article will be made available by the authors, without undue reservation.

## Ethics Statement

The animal study was reviewed and approved by Institutional Animal Care and Use Committee of Kyung Hee University.

## Author Contributions

HY and TC conceived and designed the study, performed experiments, analyzed data, and wrote the manuscript. YJ, JK, NN, and YS performed experiments and analyzed data. TC and SK supervised the study and wrote the manuscript. All of the authors revised and approved the final version of the manuscript.

### Conflict of Interest

The authors declare that the research was conducted in the absence of any commercial or financial relationships that could be construed as a potential conflict of interest.

## Footnotes

^1^http://serialbasics.free.fr/Serial_Cloner.html

## Supplementary Material

Supplementary Figure 1 ROS levels and translocation of Nrf2 is increased in PPT1 knockdown-cells. **(A)** ROS levels were detected by DCF-DA after 24 h of DM using FACS analysis. ^*^*p* < 0.01, compared to SC in PM. ^#^*p* < 0.05, compared to SC in PM. *p* < 0.01, compared to SC in DM. **(B)** The expression levels of Nrf2 on the indicated 5-days were evaluated by western blot analysis. Lamin-B is used as nuclear protein loading control.

Supplementary Figure 2 Bafilomycin A1 promoted lysosomal pH in muscle differentiation. Fluorescence was visualized by confocal microscopy by pH-sensitive LysoSensor DND-189 in proliferation medium, differentiation medium and treatment of bafilomycin 100, 200, and 400 nM. Scale bar: 200 μm.

## References

[ref1] Aicart-Ramos C. Valero R. A. Rodriguez-Crespo I. (2011). Protein palmitoylation and subcellular trafficking. Biochim. Biophys. Acta 1808, 2981–2994. 10.1016/j.bbamem.2011.07.009, PMID: 21819967

[ref56] Al-Sawaf O. Fragoulis A. Rosen C. Keimes N. Liehn E. A. Holzle F.. (2014). Nrf2 augments skeletal muscle regeneration after ischaemia-reperfusion injury. J. Pathol. 234, 538–547. 10.1002/path.4418, PMID: 25111334

[ref2] Arnold H. H. Winter B. (1998). Muscle differentiation: more complexity to the network of myogenic regulators. Curr. Opin. Genet. Dev. 8, 539–544. 10.1016/s0959-437x(98)80008-7, PMID: 9794824

[ref3] Azad M. B. Chen Y. Gibson S. B. (2009). Regulation of autophagy by reactive oxygen species (ROS): implications for cancer progression and treatment. Antioxid. Redox Signal. 11, 777–790. 10.1089/ars.2008.2270, PMID: 18828708

[ref4] Bagh M. B. Peng S. Chandra G. Zhang Z. Singh S. P. Pattabiraman N.. (2017). Misrouting of v-ATPase subunit V0a1 dysregulates lysosomal acidification in a neurodegenerative lysosomal storage disease model. Nat. Commun. 8:14612. 10.1038/ncomms14612, PMID: 28266544 PMC5344305

[ref5] Ballabio A. Gieselmann V. (2009). Lysosomal disorders: from storage to cellular damage. Biochim. Biophys. Acta 1793, 684–696. 10.1016/j.bbamcr.2008.12.001, PMID: 19111581

[ref6] Barbieri E. Sestili P. (2012). Reactive oxygen species in skeletal muscle signaling. J. Signal. Transduct. 2012:982794. 10.1155/2012/982794, PMID: 22175016 PMC3235811

[ref63] Bechet D. M. Ferrara M. J. Mordier S. B. Roux M. P. Deval C. D. Obled A. (1991). Expression of lysosomal cathepsin B during calf myoblast-myotube differentiation. Characterization of a cDNA encoding bovine cathepsin B. J. Biol. Chem. 266, 14104–14112. PMID: 1856234

[ref66] Bellezza I. Riuzzi F. Chiappalupi S. Arcuri C. Giambanco I. Sorci G.. (2020). Reductive stress in striated muscle cells. Cell Mol. Life Sci. 77, 3547–3565. 10.1007/s00018-020-03476-0, PMID: 32072237 PMC11105111

[ref7] Camp L. A. Hofmann S. L. (1993). Purification and properties of a palmitoyl-protein thioesterase that cleaves palmitate from H-Ras. J. Biol. Chem. 268, 22566–22574. PMID: 7901201

[ref62] Colella R. Roisen F. J. Bird J. W. (1986). mRNA levels of cathepsins B and D during myogenesis. Biomed. Biochim. Acta 45, 1413–1419. PMID: 3555467

[ref8] Covarrubias L. Hernandez-Garcia D. Schnabel D. Salas-Vidal E. Castro-Obregon S. (2008). Function of reactive oxygen species during animal development: passive or active? Dev. Biol. 320, 1–11. 10.1016/j.ydbio.2008.04.041, PMID: 18555213

[ref9] Das A. K. Lu J. Y. Hofmann S. L. (2001). Biochemical analysis of mutations in palmitoyl-protein thioesterase causing infantile and late-onset forms of neuronal ceroid lipofuscinosis. Hum. Mol. Genet. 10, 1431–1439. 10.1093/hmg/10.13.1431, PMID: 11440996

[ref10] De Vries H. E. Witte M. Hondius D. Rozemuller A. J. Drukarch B. Hoozemans J.. (2008). Nrf2-induced antioxidant protection: a promising target to counteract ROS-mediated damage in neurodegenerative disease? Free Radic. Biol. Med. 45, 1375–1383. 10.1016/j.freeradbiomed.2008.09.001, PMID: 18824091

[ref11] Draper J. M. Smith C. D. (2009). Palmitoyl acyltransferase assays and inhibitors (review). Mol. Membr. Biol. 26, 5–13. 10.1080/09687680802683839, PMID: 19152182 PMC2635919

[ref64] Ebisui C. Tsujinaka T. Kido Y. Iijima S. Yano M. Shibata H.. (1994). Role of intracellular proteases in differentiation of L6 myoblast cells. Biochem. Mol. Biol. Int. 32, 515–521. PMID: 8032318

[ref12] Ge Y. Chen J. (2012). Mammalian target of rapamycin (mTOR) signaling network in skeletal myogenesis. J. Biol. Chem. 287, 43928–43935. 10.1074/jbc.R112.406942, PMID: 23115234 PMC3527976

[ref60] Gordon S. J. V. Fenker D. E. Vest K. E. Padilla-Benavides T. (2019). Manganese influx and expression of ZIP8 is essential in primary myoblasts and contributes to activation of SOD2. Metallomics 11, 1140–1153. 10.1039/C8MT00348C, PMID: 31086870 PMC6584035

[ref13] Hamanaka R. B. Glasauer A. Hoover P. Yang S. Blatt H. Mullen A. R.. (2013). Mitochondrial reactive oxygen species promote epidermal differentiation and hair follicle development. Sci. Signal. 6:ra8. 10.1126/scisignal.2003638, PMID: 23386745 PMC4017376

[ref14] Khacho M. Clark A. Svoboda D. S. Azzi J. Maclaurin J. G. Meghaizel C.. (2016). Mitochondrial dynamics impacts stem cell identity and fate decisions by regulating a nuclear transcriptional program. Cell Stem Cell 19, 232–247. 10.1016/j.stem.2016.04.015, PMID: 27237737

[ref15] Kim J. Choi T. G. Ding Y. Kim Y. Ha K. S. Lee K. H.. (2008). Overexpressed cyclophilin B suppresses apoptosis associated with ROS and Ca2+ homeostasis after ER stress. J. Cell Sci. 121, 3636–3648. 10.1242/jcs.028654, PMID: 18946027 PMC2735721

[ref16] Kim J. H. Choi T. G. Park S. Yun H. R. Nguyen N. N. Y. Jo Y. H.. (2018). Mitochondrial ROS-derived PTEN oxidation activates PI3K pathway for mTOR-induced myogenic autophagy. Cell Death Differ. 25, 1921–1937. 10.1038/s41418-018-0165-9, PMID: 30042494 PMC6219511

[ref17] Kim J. Guan K. L. (2019). mTOR as a central hub of nutrient signalling and cell growth. Nat. Cell Biol. 21, 63–71. 10.1038/s41556-018-0205-1, PMID: 30602761

[ref61] Kirschke H. Wood L. Roisen F. J. Bird J. W. C. (1983). Activity of lysosomal cysteine proteinase during differentiation of rat skeletal muscle. Biochem. J. 214, 871–877. 10.1042/bj2140871, PMID: 6354179 PMC1152326

[ref18] Koster K. P. Yoshii A. (2019). Depalmitoylation by palmitoyl-protein thioesterase 1 in neuronal health and degeneration. Front. Synaptic Neurosci. 11:25. 10.3389/fnsyn.2019.00025, PMID: 31555119 PMC6727029

[ref19] Kousi M. Lehesjoki A. E. Mole S. E. (2012). Update of the mutation spectrum and clinical correlations of over 360 mutations in eight genes that underlie the neuronal ceroid lipofuscinoses. Hum. Mutat. 33, 42–63. 10.1002/humu.21624, PMID: 21990111

[ref58] Kuwahara H. Horie T. Ishikawa S. Tsuda C. Kawakami S. Noda Y.. (2010). Oxidative stress in skeletal muscle causes severe disturbance of exercise activity without muscle atrophy. Free Radic. Biol. Med. 48, 1252–1262. 10.1016/j.freeradbiomed.2010.02.011, PMID: 20156551

[ref20] Lawrence R. E. Zoncu R. (2019). The lysosome as a cellular Centre for signalling, metabolism and quality control. Nat. Cell Biol. 21, 133–142. 10.1038/s41556-018-0244-7, PMID: 30602725

[ref21] Lee J. Choi K. J. Lim M. J. Hong F. Choi T. G. Tak E.. (2010). Proto-oncogenic H-Ras, K-Ras, and N-Ras are involved in muscle differentiation via phosphatidylinositol 3-kinase. Cell Res. 20, 919–934. 10.1038/cr.2010.92, PMID: 20603646

[ref22] Lee S. Tak E. Lee J. Rashid M. A. Murphy M. P. Ha J.. (2011). Mitochondrial H2O2 generated from electron transport chain complex I stimulates muscle differentiation. Cell Res. 21, 817–834. 10.1038/cr.2011.55, PMID: 21445095 PMC3203677

[ref59] Lustgarten M. S. Jang Y. C. Liu Y. Qi W. Qin Y. Dahia P. L.. (2011). MnSOD deficiency results in elevated oxidative stress and decreased mitochondrial function but does not lead to muscle atrophy during aging. Aging Cell 10, 493–505. 10.1111/j.1474-9726.2011.00695.x, PMID: 21385310 PMC3094473

[ref23] Macauley S. L. Wozniak D. F. Kielar C. Tan Y. Cooper J. D. Sands M. S. (2009). Cerebellar pathology and motor deficits in the palmitoyl protein thioesterase 1-deficient mouse. Exp. Neurol. 217, 124–135. 10.1016/j.expneurol.2009.01.022, PMID: 19416667 PMC2679857

[ref24] Mizushima N. Levine B. (2010). Autophagy in mammalian development and differentiation. Nat. Cell Biol. 12, 823–830. 10.1038/ncb0910-823, PMID: 20811354 PMC3127249

[ref25] Molkentin J. D. Olson E. N. (1996). Defining the regulatory networks for muscle development. Curr. Opin. Genet. Dev. 6, 445–453. 10.1016/s0959-437x(96)80066-9, PMID: 8791524

[ref26] Napolitano G. Ballabio A. (2016). TFEB at a glance. J. Cell Sci. 129, 2475–2481. 10.1242/jcs.146365, PMID: 27252382 PMC4958300

[ref27] Napolitano G. Esposito A. Choi H. Matarese M. Benedetti V. Di Malta C.. (2018). mTOR-dependent phosphorylation controls TFEB nuclear export. Nat. Commun. 9:3312. 10.1038/s41467-018-05862-6, PMID: 30120233 PMC6098152

[ref28] Nita D. A. Mole S. E. Minassian B. A. (2016). Neuronal ceroid lipofuscinoses. Epileptic Disord. 18, 73–88. 10.1684/epd.2016.0844, PMID: 27629553

[ref29] Nnah I. C. Wang B. Saqcena C. Weber G. F. Bonder E. M. Bagley D.. (2019). TFEB-driven endocytosis coordinates MTORC1 signaling and autophagy. Autophagy 15, 151–164. 10.1080/15548627.2018.1511504, PMID: 30145926 PMC6287686

[ref65] Ogasawara S. Cheng X. W. Inoue A. Hu L. Piao L. Yu C.. (2018). Cathepsin K activity controls cardiotoxin-induced skeletal muscle repair in mice. J. Cachexia. Sarcopenia Muscle 9, 160–175. 10.1002/jcsm.12248, PMID: 29058826 PMC5803616

[ref30] Ownby C. L. Fletcher J. E. Colberg T. R. (1993). Cardiotoxin 1 from cobra (*Naja naja atra*) venom causes necrosis of skeletal muscle in vivo. Toxicon 31, 697–709. 10.1016/0041-0101(93)90376-t, PMID: 8342169

[ref31] Perera R. M. Zoncu R. (2016). The lysosome as a regulatory hub. Annu. Rev. Cell Dev. Biol. 32, 223–253. 10.1146/annurev-cellbio-111315-125125, PMID: 27501449 PMC9345128

[ref32] Piao Y. J. Seo Y. H. Hong F. Kim J. H. Kim Y. J. Kang M. H.. (2005). Nox 2 stimulates muscle differentiation via NF-kappaB/iNOS pathway. Free Radic. Biol. Med. 38, 989–1001. 10.1016/j.freeradbiomed.2004.11.011, PMID: 15780757

[ref57] Rajasekaran N. S. Shelar S. B. Jones D. P. Hoidal J. R. (2020). Reductive stress impairs myogenic differentiation. Redox Biol. 34:101492. 10.1016/j.redox.2020.101492, PMID: 32361680 PMC7199008

[ref33] Rebecca V. W. Nicastri M. C. Fennelly C. Chude C. I. Barber-Rotenberg J. S. Ronghe A.. (2019). PPT1 promotes tumor growth and is the molecular target of chloroquine derivatives in cancer. Cancer Discov. 9, 220–229. 10.1158/2159-8290.CD-18-0706, PMID: 30442709 PMC6368875

[ref34] Rebecca V. W. Nicastri M. C. Mclaughlin N. Fennelly C. Mcafee Q. Ronghe A.. (2017). A unified approach to targeting the lysosome’s degradative and growth signaling roles. Cancer Discov. 7, 1266–1283. 10.1158/2159-8290.CD-17-0741, PMID: 28899863 PMC5833978

[ref35] Roczniak-Ferguson A. Petit C. S. Froehlich F. Qian S. Ky J. Angarola B.. (2012). The transcription factor TFEB links mTORC1 signaling to transcriptional control of lysosome homeostasis. Sci. Signal. 5:ra42. 10.1126/scisignal.2002790, PMID: 22692423 PMC3437338

[ref36] Sandri M. (2010). Autophagy in skeletal muscle. FEBS Lett. 584, 1411–1416. 10.1016/j.febslet.2010.01.056, PMID: 20132819

[ref37] Sardiello M. Palmieri M. Di Ronza A. Medina D. L. Valenza M. Gennarino V. A.. (2009). A gene network regulating lysosomal biogenesis and function. Science 325, 473–477. 10.1126/science.1174447, PMID: 19556463

[ref38] Saxton R. A. Sabatini D. M. (2017a). mTOR signaling in growth, metabolism, and disease. Cell 169, 361–371. 10.1016/j.cell.2017.03.035, PMID: 28388417

[ref39] Saxton R. A. Sabatini D. M. (2017b). mTOR signaling in growth, metabolism, and disease. Cell 168, 960–976. 10.1016/j.cell.2017.02.004, PMID: 28283069 PMC5394987

[ref40] Settembre C. De Cegli R. Mansueto G. Saha P. K. Vetrini F. Visvikis O.. (2013). TFEB controls cellular lipid metabolism through a starvation-induced autoregulatory loop. Nat. Cell Biol. 15, 647–658. 10.1038/ncb2718, PMID: 23604321 PMC3699877

[ref41] Settembre C. Di Malta C. Polito V. A. Garcia Arencibia M. Vetrini F. Erdin S.. (2011). TFEB links autophagy to lysosomal biogenesis. Science 332, 1429–1433. 10.1126/science.1204592, PMID: 21617040 PMC3638014

[ref42] Shen H. M. Mizushima N. (2014). At the end of the autophagic road: an emerging understanding of lysosomal functions in autophagy. Trends Biochem. Sci. 39, 61–71. 10.1016/j.tibs.2013.12.001, PMID: 24369758

[ref43] Simonati A. Tessa A. Bernardina B. D. Biancheri R. Veneselli E. Tozzi G.. (2009). Variant late infantile neuronal ceroid lipofuscinosis because of CLN1 mutations. Pediatr. Neurol. 40, 271–276. 10.1016/j.pediatrneurol.2008.10.018, PMID: 19302939

[ref44] Surendran K. Vitiello S. P. Pearce D. A. (2014). Lysosome dysfunction in the pathogenesis of kidney diseases. Pediatr. Nephrol. 29, 2253–2261. 10.1007/s00467-013-2652-z, PMID: 24217784 PMC4018427

[ref45] Thada V. Miller J. N. Kovacs A. D. Pearce D. A. (2016). Tissue-specific variation in nonsense mutant transcript level and drug-induced read-through efficiency in the Cln1(R151X) mouse model of INCL. J. Cell. Mol. Med. 20, 381–385. 10.1111/jcmm.12744, PMID: 26648046 PMC4727554

[ref46] Tormos K. V. Anso E. Hamanaka R. B. Eisenbart J. Joseph J. Kalyanaraman B.. (2011). Mitochondrial complex III ROS regulate adipocyte differentiation. Cell Metab. 14, 537–544. 10.1016/j.cmet.2011.08.007, PMID: 21982713 PMC3190168

[ref47] Van Diggelen O. P. Keulemans J. L. Winchester B. Hofman I. L. Vanhanen S. L. Santavuori P.. (1999). A rapid fluorogenic palmitoyl-protein thioesterase assay: pre- and postnatal diagnosis of INCL. Mol. Genet. Metab. 66, 240–244. 10.1006/mgme.1999.2809, PMID: 10191108

[ref48] Vega-Rubin-De-Celis S. Pena-Llopis S. Konda M. Brugarolas J. (2017). Multistep regulation of TFEB by MTORC1. Autophagy 13, 464–472. 10.1080/15548627.2016.1271514, PMID: 28055300 PMC5361595

[ref49] Vesa J. Hellsten E. Verkruyse L. A. Camp L. A. Rapola J. Santavuori P.. (1995). Mutations in the palmitoyl protein thioesterase gene causing infantile neuronal ceroid lipofuscinosis. Nature 376, 584–587. 10.1038/376584a0, PMID: 7637805

[ref50] Won S. J. Cheung See Kit M. Martin B. R. (2018). Protein depalmitoylases. Crit. Rev. Biochem. Mol. Biol. 53, 83–98. 10.1080/10409238.2017.1409191, PMID: 29239216 PMC6009847

[ref51] Wu J. Lipinski M. M. (2019). Autophagy in neurotrauma: good, bad, or dysregulated. Cell 8:693. 10.3390/cells8070693, PMID: 31295858 PMC6678153

[ref52] Yoon M. S. (2017). mTOR as a key regulator in maintaining skeletal muscle mass. Front. Physiol. 8:788. 10.3389/fphys.2017.00788, PMID: 29089899 PMC5650960

[ref53] Zeidman R. Jackson C. S. Magee A. I. (2009). Protein acyl thioesterases (review). Mol. Membr. Biol. 26, 32–41. 10.1080/09687680802629329, PMID: 19115143

[ref54] Zhang M. M. Hang H. C. (2017). Protein S-palmitoylation in cellular differentiation. Biochem. Soc. Trans. 45, 275–285. 10.1042/BST20160236, PMID: 28202682 PMC5310721

[ref55] Zhang J. Wang X. Vikash V. Ye Q. Wu D. Liu Y.. (2016). ROS and ROS-mediated cellular signaling. Oxidative Med. Cell. Longev. 2016:4350965. 10.1155/2016/4350965, PMID: 26998193 PMC4779832

