# Supplementary figures and images for: Palmitoyl Protein Thioesterase 1 Is Essential for Myogenic Autophagy of C2C12 Skeletal Myoblast
Source: Front Physiol. 2020 Oct 15;11:569221. doi: 10.3389/fphys.2020.569221 (PMC7593845; doi:10.3389/fphys.2020.569221)

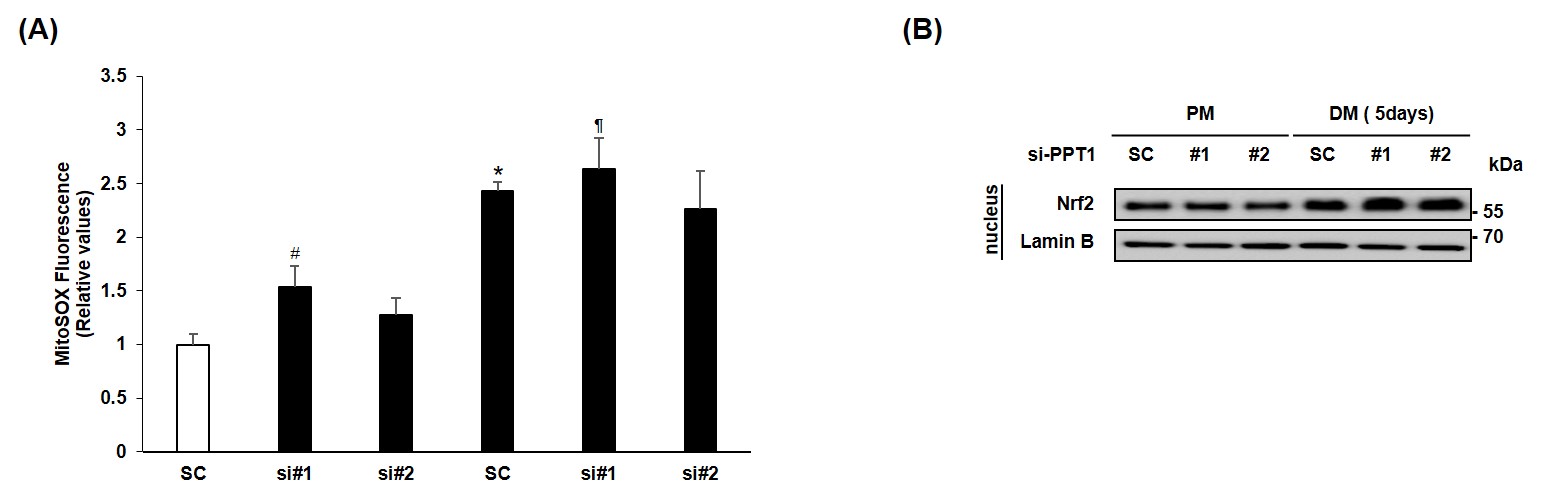

Supplement: Supplementary Figure 1 — ROS levels and translocation of Nrf2 is increased in PPT1 knockdown-cells. (A) ROS levels were detected by DCF-DA after 24 h of DM using FACS analysis. *p < 0.01, compared to SC in PM. #p < 0.05, compared to SC in PM. p < 0.01, compared to SC in DM. (B) The expression levels of Nrf2 on the indicated 5-days were evaluated by western blot analysis. Lamin-B is used as nuclear protein loading control. [file Image_1.jpeg]

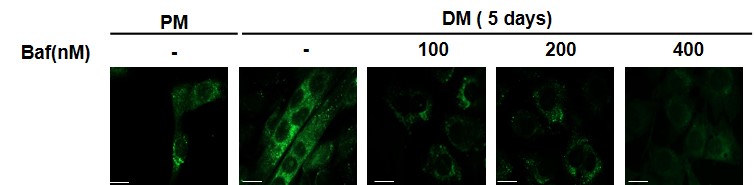

Supplement: Supplementary Figure 2 — Bafilomycin A1 promoted lysosomal pH in muscle differentiation. Fluorescence was visualized by confocal microscopy by pH-sensitive LysoSensor DND-189 in proliferation medium, differentiation medium and treatment of bafilomycin 100, 200, and 400 nM. Scale bar: 200 μm. [file Image_2.jpg]
